# Association Between Triage Score and Resource Use for Children Presenting to the Emergency Department with Behavioral Health Conditions

**DOI:** 10.1016/j.acepjo.2026.100490

**Published:** 2026-09-01

**Authors:** Ashley A. Foster, Cora H. Ormseth, Jacqueline Grupp-Phelan, Sarah J. Lowry, Elizabeth R. Alpern, Jennifer A. Hoffmann

**Affiliations:** 1 Department of Emergency Medicine, University of California, San Francisco, San Francisco, California, USA; 2 Biostatistics, Epidemiology, and Analytics for Research Core, Seattle Children’s Research Institute, Seattle, Washington, USA; 3 Division of Emergency Medicine, Ann & Robert H. Lurie Children’s Hospital of Chicago. Departments of Pediatrics and Medical Social Sciences, Northwestern University Feinberg School of Medicine. Chicago, Illinois, USA

**Keywords:** triage, pediatric emergency medicine, mental health, behavioral symptoms, pediatrics

## Abstract

**Objective:**

Among pediatric emergency department (ED) visits for behavioral health (BH), we examined the association between Emergency Severity Index (ESI) level and (1) the time to first provider evaluation and (2) ED resource use.

**Methods:**

This retrospective cross-sectional study, using the National Hospital Ambulatory Medical Care Survey, assessed ED visits of children aged 5 to 17 years. For each ESI level, we compared resource use among BH versus non-BH visits. Among BH visits, regression models examined the association of ESI level and (1) the time to first provider evaluation and (2) ED resource use, adjusted for visit characteristics.

**Results:**

There were an estimated 3,646,060 pediatric BH ED visits and 52,499,554 non-BH ED visits. Across all visits with ESI 4 to 5 (least acute), BH visits were more likely to use >1 resource (32.1%) compared with non-BH visits (15.6%; difference 16.5%, 95% confidence interval [CI] 2.0 to 30.9). Among BH visits, the most frequent ESI level was 1 to 2 (most acute; 48.5%); of these, 57.8% resulted in discharge/other disposition. Among BH visits, ESI was not associated with time to first provider evaluation; the estimated incidence rate ratio for number of different resources used was 1.59 (95% CI 1.18 to 2.14) for ESI 1 to 2 and 1.37 (95% CI 1.02 to 1.85) for ESI 3 compared with ESI 4 to 5.

**Conclusions:**

Among pediatric BH ED visits, ESI level was associated with ED resource use, but not time to first provider evaluation. Many high acuity BH visits resulted in discharge, whereas one third of low acuity BH visits required >1 resource, suggesting misalignment of assigned ESI levels with resource use.

**The Bottom Line**

This retrospective study assessed whether Emergency Severity Index triage levels accurately reflected care needs for children visiting the emergency department with behavioral health concerns. Among children with behavioral health concerns, higher Emergency Severity Index acuity was associated with greater emergency department resource use but not with shorter time to provider evaluation. Many high acuity behavioral health visits were discharged, while nearly one third of low acuity visits required multiple resources. These findings suggest that Emergency Severity Index triage levels may not consistently align with the resource needs of pediatric patients presenting with behavioral health concerns.

## 1 Introduction

### 1.1 Background

Emergency department (ED) visits for children with behavioral health (BH) emergencies, including mental health conditions and symptoms related to substance use, are rapidly increasing.^1^, ^2^, ^3^, ^4^ This poses a challenge for EDs, as demand for acute psychiatric services outstrips available resources.^5^ As a result, children with BH crises often experience prolonged ED wait times, higher rates of adverse events, such as medication errors, and patients leaving before evaluation.^6^, ^7^, ^8^, ^9^, ^10^ As the number of children presenting to EDs for BH emergencies continues to rise, the need for an accurate triage system to allocate limited ED resources based on clinical priority becomes increasingly critical.^11^ An effective triage system should facilitate rapid evaluation and initiation of interventions for high acuity patients, while also identifying which children can safely wait for care.^12^

### 1.2 Importance

Currently, most hospitals in the United States (US) use the Emergency Severity Index (ESI), a triage system with assigned levels ranging from 1 (most acute) to 5 (least acute).^13^ In addition to assessing acuity, ESI assigns triage levels partly based on anticipated ED resource utilization. Although this system has been validated for use in medical and traumatic conditions, it was not designed specifically for pediatric BH ED visits.^14^ Additionally, there is little data on ESI application to pediatric BH ED visits or alignment of the assigned ESI level with resource utilization during the ED visit.

### 1.3 Goals of This Investigation

Therefore, we sought to examine the association of ESI level with time to first provider evaluation for BH ED visits and non-BH visits among children, respectively. For BH ED visits, we aimed to assess the association between ESI level and number of different ED resources used. We hypothesized that higher acuity ESI level would be associated with greater ED resource utilization among BH visits.

## 2 Methods

### 2.1 Study Design and Data Source

We performed a retrospective cross-sectional analysis of pediatric visits to US EDs, utilizing data from the National Hospital Ambulatory Medical Care Survey (NHAMCS) from 2017-2021. NHAMCS is a nationally representative survey of outpatient and ED visits to nonfederal US hospitals conducted by the National Center for Health Statistics.^15^ The survey uses a 3-stage probability sampling design to generate nationally representative estimates from publicly available, deidentified data. This study was reviewed by the senior author’s Institutional Review Board and was determined not to meet criteria for human subjects’ research.

### 2 Selection of Included Visits

2

We included BH and non-BH ED visits by patients 5 to 17 years of age. We defined BH visits based on the presence of specific “reason for visit” (chief complaint) codes reflecting information available at triage (Table A.1),^4^ with all others considered non-BH visits. We excluded visits with missing ESI or time to first provider evaluation; visits with a length of stay of 0 minutes; and visits of patients who were dead on arrival, died in the ED, left without being seen, left before treatment was complete, or left against medical advice (Figure A.1).

### 2.3 Measurements

The exposure variable was ESI level, collapsed into 3 categories to ensure adequate sample size and stable estimates within each analytic group: immediate/emergent (1 or 2), urgent (3), and semiurgent/nonurgent (4 or 5). Visit characteristics included: age group in years (5-9, 10-14, 15-17), sex (male, female), insurance (private, public, self-pay/other), US Census Region (Midwest, Northeast, South, West), and race and ethnicity (Hispanic, non-Hispanic White, and non-Hispanic other). The non-Hispanic other category included Asian, Native Hawaiian or Other Pacific Islander, American Indian or Alaska Native, non-Hispanic Black, and 2 or more races, which were combined due to small sample size within each group.

Clinical visit characteristics included: season of visit (school month versus non-school month), weekday versus weekend, time of visit, length of visit in minutes, arrival mode by ambulance, presence of a co-occurring medical reason for visit, and disposition. The season of visit of school month was defined as months September through May to reflect previously reported seasonal trends of pediatric BH ED visits.^16^ Time of visit was defined as daytime (7 AM - 3 PM), evening (3 PM -11 PM), and overnight (11 PM - 7 AM). Presence of a co-occurring medical reason for visit was defined as the presence of any non-BH reason for visit code. Disposition was classified as admission or transfer (representing admission to the hospital, admission to an observation unit, or transfer to another facility), or discharge/other disposition.

### 2.4 Outcomes

The primary outcome variables were time to first provider evaluation and number of different resources used during the ED visit. Time to first provider evaluation was defined as the time in minutes between ED arrival and being seen by an ED clinician (physician, nurse practitioner, or physician assistant). The number of different resources used was aligned with ESI handbook recommendations, with each of the following considered a distinct resource (1 point each): laboratory tests, electrocardiogram, radiograph, advanced imaging, intravenous fluids, nebulized therapy, specialty consultation, and simple procedure; 2 points were assigned for a complex procedure (Table A 2).^17^ The ESI handbook recommends that triage nurses assign an ESI level of 3, 4, and 5 to visits expected to have ≥1, 1, and 0 different resources used, respectively, if vitals are within normal ranges and no life-threatening or high-risk conditions are identified.

### 2.5 Analysis

A complete case analysis was used, including only visits with no missing data for the relevant variables. We described characteristics of BH ED visits and non-BH ED visits by ESI level. We utilized NHAMCS estimation procedures, which account for multistage complex survey sampling techniques to produce national visit estimates.^18^ We compared BH and non-BH visits across ESI levels in terms of number of different resources used, length of visit, disposition, and time to first provider evaluation, estimating percentages, means, and medians with 95% CI (Stata package epctile).^19^^,^^20^

Given the right-skewed distribution of wait time, we used generalized linear models with a log link and gamma family to estimate the relative difference in wait time to first provider evaluation associated with ESI level. ESI level was modeled as a categorical variable with ESI 4–5 as the reference. We adjusted for selected visit characteristics, with separate models for BH visits and non-BH visits. Visit characteristics were decided a priori based on prior literature and clinical characteristics that may influence ED triage: age, sex, race and ethnicity, insurance, co-occurring medical diagnosis, US Census Region, time of visit, day of week of visit, season of visit, and year.^14^^,^^21^, ^22^, ^23^, ^24^ Results were reported as exponentiated coefficients with 95% CIs, interpreted as ratios relative to the reference group.

Among BH visits, we used negative binomial regression to estimate the number of different resources used associated with ESI level, adjusted for the same visit characteristics. Results were reported as incidence rate ratios (IRR) with 95% CIs. Reference groups were chosen to reflect the largest category within the sample, with exception of ESI (reference group: ESI 4-5), which was chosen based on clinical relevance. Analyses were performed using Stata version 18.0 (StataCorp, College Station, TX).

## 3 Results

### 3.1 Characteristics of Study Subjects

From 2017-2021, there were an estimated 3,646,060 (7.4%) pediatric BH ED visits and 52,499,554 (92.6%) pediatric non-BH ED visits nationally (Table 1). Forty-two percent of pediatric BH visits were by children 10-14 years old, 62.7% were female, and 54.1% were publicly insured. Forty percent of non-BH visits were by children 5-9 years old, 50.9% were female, and 68.0% were publicly insured. Of pediatric BH visits, 48.5% were designated as ESI 1-2, 31.9% ESI 3, and 19.6% ESI 4-5. Among non-BH visits, 9.1% were assigned an ESI 1-2, 37.6% an ESI 3, and 53.3% an ESI 4-5. Sixty-two percent of pediatric BH visits had a co-occurring medical reason for visit. Among all pediatric ED visits, 61.6% of BH visits and 94.2% of non-BH visits resulted in discharge from the ED. Of BH visits with an ESI of 1-2, 57.8% resulted in discharge or other disposition (Table 2).

**Table 1:** Characteristics of pediatric behavioral health and nonbehavioral health emergency department visits, 2017-2021.

	Behavioral Health Visit, Weighted N	Percent Estimate (95% Confidence Interval)	Nonbehavioral Health Visit, Weighted N	Percent Estimate (95% Confidence Interval)
	n = 3,646,060^a^		n = 52,499,554^a^	
Age (years)				
5-9	550,954	15,1 (11.1 - 20.3)	21,195,643	40.4 (37.9 - 42.9)
10-14	1,517,270	41.6 (35.0 - 48.5)	18,345,649	34.9 (33.1 - 36.8)
15-17	1,577,836	43.3 (37.0 - 49.8)	12,958,261	24.7 (22.9 - 26.5)
Sex				
Female	2,284,324	62.7 (56.7 - 68.2)	26,728,069	50.9 (48.9 - 52.9)
Male	1,361,736	37.3 (31.8 - 43.3)	25,771,485	49.1 (47.1 - 51.1)
Race and Ethnicity				
Hispanic	804,094	22.1 (15.7 - 30.0)	15,322,336	29.2 (25.1 - 33.7)
Non-Hispanic Other	815,037	22.4 (17.1 - 28.6)	14,089,782	26.8 (24.3 - 29.5)
Non-Hispanic White	2,026,929	55.6 (47.4 - 63.5)	23,087,436	44.0 (40.1 - 47.9)
Insurance				
Private	1,294,305	38.4 (30.7 - 46.7)	12,382,021	25.5 (23.4 - 27.7)
Public	1,823,413	54.1 (46.2 - 61.9)	33,016,556	68.0 (65.4 - 70.5)
Self-pay/Other	251,005	7.5 (4.3 - 12.5)	3,165,801	6.5 (5.3 - 7.9)
Co-occurring Medical Reason for Visit	2,245,074	61.6 (54.3 - 68.4)	NA	NA
U.S. Census Region				
Midwest	898,903	24.7 (17.4 - 33.7)	11,360,476	21.6 (16.9 - 27.3)
Northeast	792,589	21.7 (14.8 - 30.8)	8.259,600	15.7 (12.0 - 20.3)
South	1,291,820	35.4 (25.9, 46.3)	22,435,767	42.7 (35.3 - 50.5)
West	662,748	18.2 (12.3 - 26.1)	10,443,711	19.9 (15.3 - 25.5)
Arrival by Ambulance^b^	958,738	27.7 (21.3 - 35.1)	3,457,929	6.8 (4.7 - 9.6)
Emergency Severity Index (ESI)				
1-2	1,767, 788	48.5 (40.6 - 56.5)	4,765,083	9.1 (6.9 - 11.9)
3	1,164,916	31.9 (26.0 - 38.6)	19,739,181	37.6 (34.6 -40.7)
4-5	713,356	19.6 (14.6 - 25.7)	27,995,289	53.3 (49.1 -57.5)
Time of Visit				
Daytime (7:00 AM - 3:00 PM)	1,323,430	36.3 (29.8 - 43.3)	19,727,827	37.6 (35.7 - 39.5)
Evening (3:00 PM -11:00 PM)	1,710,585	46.9 (41.0 - 52.9)	26,207,849	49.9 (48.0 - 51.8)
Overnight (11:00 PM - 7:00 AM)	612,046	16.8 (12.6 - 22.0)	6,563,878	12.5 (11.0 - 14.1)
Day of Week of Visit				
Weekday (Monday-Friday)	2,762,087	75.8 (70.3 - 80.5)	37,992,893	72.4 (70.9 - 73.8)
Weekend (Saturday-Sunday)	883,973	24.2 (19.5 - 29.7)	14,506,661	27.6 (26.2 - 29.1)
Season of Visit				
Non-School Month (June-August)	664,024	18.2 (12.5 - 25.7)	10,597,111	20.2 (15.7 - 25.5)
School Month (September-May)	2,982,036	81.8 (74.3 - 87.5)	41,902,443	79.8 (74.5 - 84.3)
Disposition				
Admission or Transfer	1,078,832	29.6 (22.5 - 37.8)	3,033,669	5.8 (3.9 - 8.4)
Discharge or Other	2,567,229	61.6 (62.2 - 77.5)	49,465,884	94.2 (91.6 - 96.1)

a Unweighted behavioral health visit n = 504; unweighted nonbehavioral health visit = 6302.

b Data missingness: 576 visits are missing data on Payer Type (8.5% overall; 9.5% in Behavioral Health visits and 8.4% in Nonbehavioral Health visits); 170 visits (2.5%) are missing data on Arrival by ambulance (3.6% in Behavioral Health visits and 2.4% in Nonbehavioral health visits).

**Table 2:** Markers of resource utilization across emergency severity index levels during pediatric behavioral health and non-behavioral health emergency department visits.

Behavioral Health Visits			
	Emergency Severity Index 1-2	Emergency Severity Index 3	Emergency Severity Index 4 - 5
	Weighted, n = 1,767,788	Weighted, n = 1,164,916	Weighted, n = 713,356
Number of Different Resources Used, percentage (95% CI)			
0	a	19.0 (12.0 - 28.7)	37.0 (25.4 - 50.3)
1	34.7 (25.4 - 45.2)	28.3 (19.9 - 38.5)	30.9 (21.6 - 42.1)
>1	54.2 (42.7 - 65.2)	52.7 (41.6 - 63.6)	32.1 (19.7 - 47.7)
Length of Visit in Minutes,^b^ median (95% CI)	263 (156 - 370)	188 (153-223)	124 (107 - 141)
Disposition, percentage (95% CI)			
Admission or Transfer	42.2 (31.7 - 53.5)	26.5 (17.4 - 38.3)	a
Discharge or Other	57.8 (46.5 - 68.3)	73.5 (61.7 - 82.6)	96.7 (90.7 - 98.9)

Non-Behavioral Health Visits			
	Emergency Severity Index 1 - 2	Emergency Severity Index 3	Emergency Severity Index 4-5
	Weighted, n = 4,765,083	Weighted, n = 19,739,181	Weighted, n = 27,995,289
Number of Different Resources Used, percentage (95% CI)			
0	15.1 (10.8 - 20.8)	21.6 (18.9 - 24.6)	43.6 (40.6 - 46.7)
1	24.5 (20.0 - 29.6)	32.3 (29.8 - 35.0)	40.7 (38.1 - 43.4)
>1	60.4 (52.2 - 68.0)	46.0 (42.5 - 49.6)	15.6 (13.5 - 18.0)
Length of Visit in Minutes^b^^,^^c^, median (95% CI)	197 (172 - 222)	164 (152 - 176)	102 (96 - 108)
Disposition, percentage (95% CI)			
Admission or Transfer	24.0 (15.4 - 35.5)	7.8 (5.3 - 11.4)	1.2 (0.7 - 2.2)
Discharge or Other	76.0 (64.5 - 84.6)	92.2 (88.6 - 94.7)	98.8 (97.8 - 99.3)

a Suppressed due to relative standard error > 30%.

b Interquartile range (minutes): BH ESI 1-2 (165-684), BH ESI 3 (124-363), BH ESI 4-5 (84-201); Non-BH ESI 1-2 (116-290), Non-BH ESI 3 (106-238), Non-BH ESI 4-5 (69-154).

c Missing data: in the unweighted data, for Length of Visit, there is 21% missingness among BH visits, and 22% missingness among non-BH visits. Missingness by year: 2017—95.9% missing, 2018—2.2% missing, 2019—0.8% missing, 2020—0.6% missing, 2021—0.5% missing.

### 3.2 Main Results

Among visits with ESI 4-5, BH visits were more likely to use >1 different resource (32.1%) compared to non-BH visits (15.6%; difference: 16.5%; 95% CI: 2.0-30.9%). Median time to first provider evaluation was 13 minutes for BH visits and 16 minutes for non-BH visits (percent difference -3.0, 95% CI -7.5 - 1.5; Table 3). BH visits used a greater mean number of different resources (1.7) than non-BH visits (1.3; percent difference 0.38, 95% CI 0.17 – 0.58).

**Table 3:** Time to first provider evaluation and number of different resources used in behavioral health emergency department visits, overall and by Emergency Severity Index.

	Behavioral Health Visit		Non-Behavioral Health Visit		Difference (95% CI)
	Median Minutes to First Provider Evaluation (95% CI)	Interquartile Range, Minutes	Median Minutes to First Provider Evaluation (95% CI)	Interquartile Range, Minutes	
Overall	13 (8 - 18)	5-40	16 (14-19)	6-36	-3.0 (-7.5 - 1.5)
Emergency Severity Index 1-2	12 (7 - 17)	5-31	13 (9-17)	5-30	-1.0 (-7.5 – 5.5)
Emergency Severity Index 3	15 (10 - 20)	6-40	14 (11-17)	5-34	1.0 (-4.2 – 6.2)
Emergency Severity Index 4-5	20 (10 - 30)	6-41	17 (15-20)	6-39	3.0 (-7.3 – 13.3)

	Mean Number of Different Resources (95% CI)	Standard Deviation	Mean Number of Different Resources (95% CI)	Standard Deviation	
Overall	1.7 (1.5 - 1.9)	1.5	1.3 (1.2-1.4)	1.4	0.38 (0.17 – 0.58)
Emergency Severity Index 1-2	1.9 (1.6-2.2)	1.5	2.4 (2.0-2.7)	1.8	-0.45 (-0.96 – 0.05)
Emergency Severity Index 3	1.7 (1.4-2.0)	1.5	1.8 (1.7-1.9)	1.6	-0.04 (-0.34 – 0.26)
Emergency Severity Index 4-5	1.1 (0.8-1.4)	1.2	0.8 (0.8-0.9)	1.0	0.28 (-0.04 – 0.59)

In the generalized linear model that adjusted for visit-level characteristics, there was no association between ESI level and time to first provider evaluation for BH visits (Table 4). In the generalized linear model for non-BH visits, the relative difference in time to first provider was significantly shorter for patients with an ESI level 1-2 (exponentiated coefficient 0.81, 95% CI 0.67, 0.97) representing a 19% shorter wait time compared to those with ESI level 4-5.

**Table 4:** Relative differences in time to first provider evaluation by emergency severity index level, in behavioral health and nonbehavioral health emergency department visits, adjusted for visit characteristics.

	Exponentiated Coefficient (95% CI)	
	Behavioral Health Visits	Nonbehavioral Health Visits
ESI level		
1-2	0.99 (0.68, 1.46)	0.81 (0.67, 0.97)
3	0.90 (0.64, 1.26)	1.00 (0.87, 1.14)
4-5	Referent	Referent
Race and Ethnicity		
Hispanic	1.19 (0.79, 1.77)	1.17 (1.01, 1.36)
Non-Hispanic Other	0.95 (0.64, 1.41)	0.98 (0.86, 1.11)
Non-Hispanic White	Referent	Referent
Age (years)		
5 - 9	0.84 (0.56, 1.27)	0.94 (0.84, 1.06)
10 - 14	Referent	Referent
15 - 17	1.19 (0.89, 1.58)	1.01 (0.87, 1.17)
Sex		
Female	Referent	Referent
Male	1.28 (0.93, 1.77)	0.87 (0.79, 0.96)
Insurance		
Public	Referent	Referent
Private	0.94 (0.66, 1.34)	0.93 (0.83, 1.05)
Self-pay/Other	0.73 (0.45, 1.17)	0.93 (0.77, 1.12)
Co-occurring Medical Reason for Visit		
Yes	0.99 (0.70, 1.39)	(NA)
No	Referent	
US Census Region		
Midwest	1.75 (1.16, 2.65)	1.02 (0.83, 1.24)
Northeast	2.94 (1.92, 4.49)	1.46 (1.18, 1.80)
South	Referent	Referent
West	1.61 (1.00, 2.61)	1.12 (0.85, 1.46)
Time of Visit		
Daytime (7:00 AM - 3:00 PM)	0.90 (0.67, 1.21)	0.86 (0.78, 0.95)
Evening (3:00 PM -11:00 PM)	Referent	Referent
Overnight (11:00 PM - 7:00 AM)	1.60 (1.00, 2.54)	0.84 (0.71, 1.01)
Day of Week of Visit		
Weekday (Monday-Friday)	Referent	Referent
Weekend (Saturday – Sunday)	1.29 (0.96, 1.73)	0.79 (0.70, 0.88)
Season of Visit		
School Month (September-May)	Referent	Referent
Non-School Month (June-August)	0.67 (0.40, 1.13)	0.81 (0.66, 1.00)

ESI: Emergency Severity Index.

**Figure 1:**
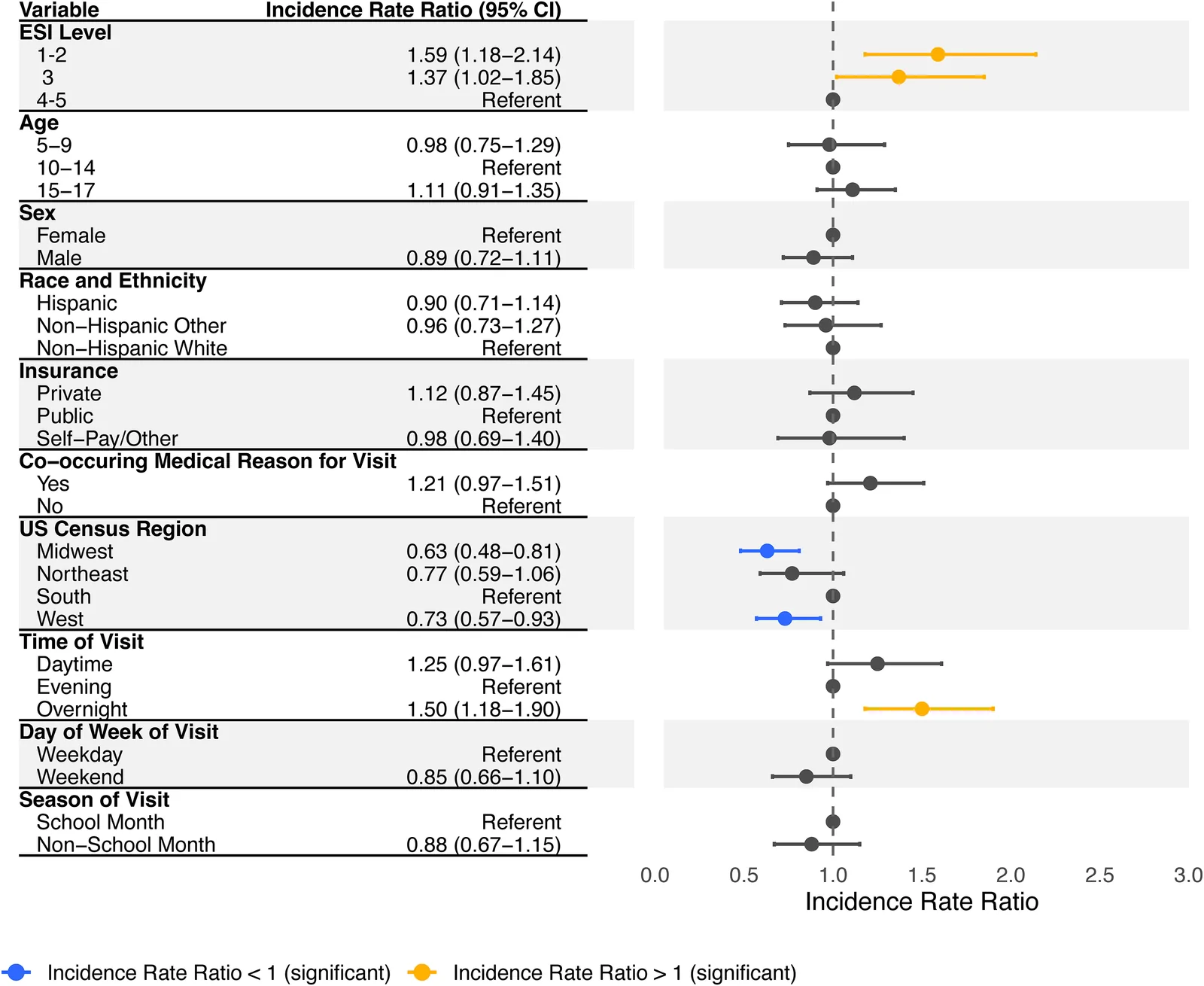
Adjusted incidence rate ratios for number of different resources used associated with emergency severity index, in pediatric behavioral health emergency department visits. Negative binomial regression analysis was used to estimate the number of different emergency department resources used associated with Emergency Severity Index level, adjusted for the following visit characteristics: age, sex, race and ethnicity, insurance, co-occurring medical reason for visit, US census region, time of visit, day of week of visit, and season of visit. Results were reported as incidence rate ratios with 95% confidence intervals (CIs).

After adjusting for visit-level characteristics, the estimated number of different resources used during BH visits with ESI 4-5 (the referent group) was 1.31 (95% CI 0.80, 2.12) (Figure 1). The estimated IRR for the number of different resources used in BH visits with ESI 1-2 was 1.59 (95% CI 1.18, 2.14) and for ESI 3 was 1.37 (95% CI 1.02, 1.85), compared to visits with ESI 4-5. There were no statistically significant differences in estimated number of resources used by age, sex, race and ethnicity, insurance, or co-occurring medical reason for visit. BH visits occurring in the Midwest (IRR 0.63; 95% CI 0.48, 0.81) and the West (IRR 0.73; 95% CI 0.57,0.93) had a lower number of different resources used compared to visits in the South. Overnight BH visits had a greater number of different resources used with an IRR 1.50 (95% CI 1.18, 1.90) compared to the evening.

## 4 Limitations

NHAMCS is subject to practice and coding variation across institutions and does not capture medication route, vital signs, Glasgow Coma Scale, or the nurse rationale for triage assignment. Consequently, some ESI-designated resources and factors used to assign ESI levels could not be evaluated. Additionally, ESI 1-2 designations are based on high-risk features rather than predicted resource use, and several of these features, as well as other markers of illness severity (e.g., acute agitation requiring pharmacologic restraint), are unavailable in NHAMCS and may represent unmeasured confounders. Results should be interpreted in the context of missing data for certain study variables, which resulted in exclusion of a subset of visits from the analytic sample. While these exclusions may have affected generalizability, the extent and direction of any resulting biases are uncertain. Finally, the study period encompassed the COVID-19 pandemic, during which changes in ED utilization and operations may have influenced triage practices and resource utilization.

## 5 Discussion

This retrospective cross-sectional analysis of geographically representative visits to US EDs found that, during pediatric BH ED visits, there was a statistically significant association between ESI level and resource use, with greater resource use occurring during visits with higher assigned acuity level. Differences in the distribution of triage scores were noted in BH and non-BH visits, with BH visits most frequently assigned ESI 1-2 and non-BH visits most frequently assigned ESI 4-5. Although ESI 1-2 represents the highest acuity, most BH visits triaged to ESI level 1-2 ultimately resulted in discharge to home, reflecting BH visits may require urgent evaluation yet still be amenable to stabilization and return to the community. In the adjusted analysis of BH visits, time to first provider evaluation did not significantly differ by ESI level, but for non-BH visits, time to first provider evaluation was significantly shorter for visits with ESI 1-2 compared to visits with ESI 4-5.

Among BH visits, the most frequent triage categorization was ESI 1-2, with nearly half of visits triaged to this acuity level. This finding may be explained by the ESI handbook guidelines, which recommend that patients presenting as a danger to themselves or others should be triaged as ESI 2.^25^^,^^26^ In addition, some BH visits require enhanced safety monitoring, such as continuous observation for suicide risk, representing staffing resources that are not explicitly captured within the ESI resource prediction framework. Relative to rates identified in this study, prior studies have found higher rates of high acuity ESI level among pediatric BH visits. Among children’s hospitals in the Pediatric Emergency Care Applied Research Network Registry, 61% of BH visits were triaged to ESI 2,^27^ and a retrospective single-center study at a pediatric ED reported 82% of BH visits were triaged to ESI 2.^28^ The lower proportion of patients triaged to ESI 2 in our study, primarily composed of visits to general EDs, may reflect practice variation between general and pediatric EDs.^29^ Given the majority of US children receive care in general EDs rather than children’s hospital EDs,^30^^,^^31^ it is essential to deepen our understanding of pediatric triage practices within general ED settings. However, much of pediatric research remains concentrated in children’s hospitals,^32^^,^^33^ highlighting a critical gap that must be addressed to ensure equitable and effective emergency care for all children.

Among pediatric BH ED visits assigned a high acuity level (ESI 1-2) in our study, nearly 60% of visits resulted in discharge. The overall discharge rate in our study aligns with prior research reporting pediatric BH discharge rates between 53% and 72%.^27^^,^^28^^,^^34^ Unlike many medical emergencies where high triage acuity is often associated with admission or intensive diagnostic evaluation, BH visits may require urgent assessment and stabilization yet still appropriately result in discharge to the community. As the triage process is intended to sort individuals based on the severity and urgency of their condition, this high discharge rate may also suggest the ESI categorization does not fully capture the clinical needs of BH visits. Although BH visits may be prioritized for rapid room placement due to safety concerns such as elopement prior to evaluation, this mismatch may still divert critical resources and swift attention from those patients with severe illness and injury. Furthermore, when most children with BH symptoms are assigned the same severity level (i.e., ESI 2), determining which patients should be seen first becomes a subjective process, increasing the risk of bias.^24^^,^^35^ An effective triage system should minimize subjective decision-making to reduce bias and ensure timely care for those in greatest need.

The ESI algorithm recommends assigning ESI 3, 4, and 5 to visits likely to use more than one resource, one resource, and no resources, respectively.^26^ Because predicted resource utilization is a central component of the ESI triage framework,^17^ examining actual resource use provides insight into whether ESI categorization aligns with clinical care patterns for BH visits. Our study revealed that for pediatric BH visits, zero or one different resources was used in 47.3% of visits assigned an ESI 3 and more than one different resource was used in 32.1% of visits assigned ESI 4-5. This distribution suggests imperfect alignment between resource use and ESI categorization in both directions. Prior studies have demonstrated discordance between ESI assignment and subsequent resource utilization among pediatric ED visits, including under triage rates of 7% to 23% and overtriage rates of 44% to 59%.^29^^,^^36^ Similar findings have been reported among pediatric behavioral health visits, in which 57% of encounters were over-triaged and 9% were undertriaged.^37^ Our findings suggest that pediatric BH visits may be particularly susceptible to mistriage. Compared with non-BH visits, BH ESI 1-2 visits used fewer resources, whereas BH ESI 4-5 visits used more resources. Although ESI level was associated with resource utilization among BH visits, the substantial mismatch between ESI assignment and subsequent resource use suggests opportunities to improve triage processes. Mistriage may contribute to delayed care, and worse outcomes among under-triaged patients,^38^^,^^39^ while over-triage may increase unnecessary testing and inefficient use of limited resources.^40^

During BH visits, ESI level was not associated with time to first provider evaluation. However, for non-BH visits, time to first provider evaluation was significantly shorter for ESI level 1-2 compared to ESI level 4-5. To our knowledge, this is the first study to investigate this association among pediatric BH and non-BH ED visits. While the ESI system is not designed to provide recommended wait times to see a provider, this parameter can serve as a measure of perceived illness severity by ED staff, with lower wait times for patients with higher acuity ESI levels. However, our finding of no association between ESI level and time to first provider evaluation for BH visits suggests that, in the absence of effective triage categorization of BH symptoms, clinicians may rely on their own judgment to prioritize which children with BH symptoms require rapid evaluation. Without an objective triage system, clinician judgment becomes susceptible to bias, which can lead to disparities by race, ethnicity, and preferred language that have been previously reported in pediatric triage practices.^24^^,^^37^

These findings may help inform future work on triage systems specifically tailored to BH visits, as safety-related resources such as enhanced observation for patients at risk of self-harm are not explicitly incorporated into the ESI framework. Future directions can include comparison of ESI to alternative triage models for pediatric BH visits, or integration of BH-specific modifications within the ESI framework to better account for safety monitoring and risk assessment needs. For instance, the Australian Mental Health Triage Scale (AMHTS) is widely used in Australian EDs.^41^ Among US adult patients with psychiatric symptoms, AMHTS was associated with reduction in wait times and better alignment with the recommended time to provider evaluation protocols relative to ESI.^42^ Additionally, within a children’s hospital ED, implementation of an adapted AMHTS assessment along with a bundle of workflow, staff, and physical space modifications led to decreased nurse injury rates, security code activations, patient length of stay, and BH admission rates.^12^ Because AMHTS has not been validated in pediatric ED populations, future studies should compare its performance with ESI and evaluate its association with resource utilization and patient outcomes.

In summary, this study suggests that currently used ESI algorithm in US EDs may not fully align acuity categorization with patterns of resource utilization for pediatric BH visits. While ESI level was associated with resource use in adjusted models, we also found frequent differences between the number of different resources used and those anticipated by the ESI framework. Additionally, ESI level was not associated with time to first provider evaluation for BH visits, in contrast to non-BH visits in which higher acuity ESI levels were associated with shorter time to provider evaluation. These findings suggest ED clinicians may rely more heavily on clinical judgment and safety considerations when prioritizing BH visits, which may not be fully captured within the resource-based structure of the ESI algorithm. Future studies should investigate BH-specific triage approaches in children and their relationship to ED operations and patient outcomes.

## Author Contributions

Drs. Foster and Hoffmann conceptualized and designed the study, drafted the initial manuscript, coordinated and supervised data collection, and reviewed and revised the manuscript. Dr. Lowry collected data, carried out data analysis and interpretation, and reviewed and revised the manuscript. Drs. Ormseth, Grupp-Phelan, and Alpern interpreted the data and critically reviewed and revised the manuscript. All authors have approved the final manuscript as submitted and agree to be accountable for all aspects of the work.

## Funding and Support

By *JACEP Open* policy, all authors are required to disclose any and all commercial, financial, and other relationships in any way related to the subject of this article as per ICMJE conflict of interest guidelines (see www.icmje.org). The Pediatric Pandemic Network is supported in part by the 10.13039/100000102Health Resources and Services Administration (HRSA) of the 10.13039/100000016US Department of Health and Human Services (HHS) as part of cooperative agreements U1IMC43532 and U1IMC45814 with 0 percent financed with nongovernmental sources. The content presented here is that of the authors and does not necessarily represent the official views of, nor an endorsement by HRSA, HHS, or the US Government. For more information, visit HRSA.gov. Research reported in this publication was supported by the 10.13039/100000025National Institute of Mental Health of the 10.13039/100000002National Institutes of Health under Award Number K23MH135206 [to JAH]. The content is solely the responsibility of the authors and does not necessarily represent the official views of the National Institutes of Health.

## Data Sharing Statement

All deidentified data files and data dictionary are available as of December 20, 2025 at https://www.cdc.gov/nchs/nhamcs/about/index.html.

## Conflicts of Interest

AAF receives research funding outside of the submitted work from Abbott Laboratories.

All authors have affirmed they have no conflicts of interest to declare.

## References

[1] Leyenaar J.K., Freyleue S.D., Bordogna A., Wong C., Penwill N., Bode R. (2021). Frequency and duration of boarding for pediatric mental health conditions at acute care hospitals in the US. JAMA.

[2] Leeb R.T., Bitsko R.H., Radhakrishnan L., Martinez P., Njai R., Holland K.M. (2020). Mental health–related emergency department visits among children aged 18 years during the COVID-19 pandemic — United States, January 1–October 17, 2020. MMWR Morb Mortal Wkly Rep.

[3] Santillanes G., Axeen S., Lam C.N., Menchine M. (2020). National trends in mental health-related emergency department visits by children and adults, 2009–2015. Am J Emerg Med.

[4] Bommersbach T.J., McKean A.J., Olfson M., Rhee T.G. (2023). National trends in mental health–related emergency department visits among youth, 2011-2020. JAMA.

[5] Bowden C.F., True G., Cullen S.W. (2021). Treating pediatric and geriatric patients at risk of suicide in general emergency departments: perspectives from emergency department clinical leaders. Ann Emerg Med.

[6] Sethuraman U., Kannikeswaran N., Farooqi A., Richards K., Chamberlain J. (2021). Antipsychiatric medication errors in children boarded in a pediatric emergency department. Pediatr Emerg Care.

[7] Gross T.K., Lane N.E., Timm N.L. (2023). Crowding in the emergency department: challenges and best practices for the care of children. Pediatrics.

[8] McEnany F.B., Ojugbele O., Doherty J.R., McLaren J.L., Leyenaar J.A.K. (2020). Pediatric mental health boarding. Pediatrics.

[9] Claudius I., Donofrio J.J., Lam C.N., Santillanes G. (2014). Impact of boarding pediatric psychiatric patients on a medical ward. Hosp Pediatr.

[10] Feuer V., Mooneyham G.C., Malas N.M. (2023). Addressing the pediatric mental health crisis in emergency departments in US: findings of a national pediatric boarding consensus panel. J Acad Consult Liaison Psychiatry.

[11] FitzGerald G., Jelinek G.A., Scott D., Gerdtz M.F. (2010). Emergency department triage revisited. Emerg Med J.

[12] Stricker F.R., O’Neill K.B., Merson J., Feuer V. (2018). Maintaining safety and improving the care of pediatric behavioral health patients in the emergency department. Child Adolesc Psychiatr Clin N Am.

[13] McHugh M., Tanabe P., McClelland M., Khare R.K. (2012). More patients are triaged using the emergency severity index than any other triage acuity system in the United States. Acad Emerg Med.

[14] Travers D.A., Waller A.E., Katznelson J., Agans R. (2009). Reliability and validity of the emergency severity index for pediatric triage. Acad Emerg Med.

[15] U.S. Centers for Disease Control and Prevention National hospital ambulatory medical care survey. https://www.cdc.gov/nchs/nhamcs/about/index.html.

[16] Radhakrishnan L., Carey K., Pell D. (2023). Seasonal trends in emergency department visits for mental and behavioral health conditions among children and adolescents aged 5–17 years — United States, January 2018–June 2023. MMWR Morb Mortal Wkly Rep.

[17] Gilboy N, Tanabe T, Travers D, Rosenau AM (2011). Implementation Handbook 2012 Edition. AHRQ Publication No. 12-0014.

[18] Centers for Disease Control and Prevention: National Center for Health Statistics (2015). Ambulatory health care data: NHAMCS estimation procedures. https://archive.cdc.gov/www_cdc_gov/nchs/ahcd/ahcd_estimation_procedures.htm.

[19] Francisco C., Fuller W. (1991). Quantile estimation with a complex survey design. Ann Stat.

[20] Kolenikov S. EPCTILE: Stata module to estimate percentiles with complex survey data. https://staskolenikov.net/stata/.

[21] Marshall R., Ribbers A., Sheridan D., Johnson K.P. (2021). Mental health diagnoses and seasonal trends at a pediatric emergency department and hospital, 2015-2019. Hosp Pediatr.

[22] Frankenberger W.D., Zorc J.J., Ten Have E.D., Brodecki D., Faig W.G. (2024). triage accuracy in pediatrics using the emergency severity index. J Emerg Nurs.

[23] Joseph J.W., Kennedy M., Landry A.M. (2023). Race and ethnicity and primary language in emergency department triage. JAMA Netw Open.

[24] Zook H.G., Kharbanda A.B., Flood A., Harmon B., Puumala S.E., Payne N.R. (2016). Racial differences in pediatric emergency department triage scores. J Emerg Med.

[25] Broadbent M., Jarman H., Berk M. (2002). Improving competence in emergency mental health triage. Accid Emerg Nurs.

[26] Emergency Nurses Association *Emergency Severity Index Handbook*. Fifth Edition. 2023. https://californiaena.org/wp-content/uploads/2023/05/ESI-Handbook-5th-Edition-3-2023.pdf.

[27] Hoffmann J.A., Carter C.P., Olsen C.S. (2024). Pediatric mental health emergency department visits from 2017 to 2022: a multicenter study. Acad Emerg Med.

[28] Hudgins J.D., Monuteaux M.C., Kent C. (2025). Changes in behavioral health visits, operations, and boarding in a pediatric emergency department. Ann Emerg Med.

[29] Geanacopoulos A.T., Peltz A., Melton K. (2025). Pediatric triage accuracy in pediatric and general emergency departments. Hosp Pediatr.

[30] Whitfill T., Auerbach M., Scherzer D.J., Shi J., Xiang H., Stanley R.M. (2018). Emergency care for children in the united states: epidemiology and trends over time. J Emerg Med.

[31] Gausche-Hill M., Ely M., Schmuhl P. (2015). A national assessment of pediatric readiness of emergency departments. JAMA Pediatr.

[32] McDaniel C.E., Coon E.R., Paciorkowski N. (2025). Pediatric randomized clinical trials in community hospitals: strategies to enhance site participation and engagement. Hosp Pediatr.

[33] Keren R., Luan X., Localio R. (2012). Prioritization of comparative effectiveness research topics in hospital pediatrics. Arch Pediatr Adolesc Med.

[34] Case S.D., Case B.G., Olfson M., Linakis J.G., Laska E.M. (2011). Length of stay of pediatric mental health emergency department visits in the United States. J Am Acad Child Adolesc Psychiatry.

[35] Gorski J.K., Alpern E.R., Lorenz D.J., Ramgopal S. (2023). Racial and ethnic disparities in emergency department wait times for children: analysis of a nationally representative sample. Acad Pediatr.

[36] Sax D.R., Warton E.M., Kene M.V. (2024). Emergency severity index version 4 and triage of pediatric emergency department patients. JAMA Pediatr.

[37] Hoffmann J.A., Foster A.A., Rojas C.R. (2026). Overtriage and undertriage of children presenting to the emergency department for behavioral health. JAMA Netw Open.

[38] Berkowitz D., Cohen J.S., McCollum N., Rojas C.R., Chamberlain J.M. (2023). Delays in treatment and disposition attributable to undertriage of pediatric emergency medicine patients. Am J Emerg Med.

[39] Ageron F.X., Porteaud J., Evain J.N. (2021). Effect of under triage on early mortality after major pediatric trauma: a registry-based propensity score matching analysis. World J Emerg Surg.

[40] DeKeyser F.G., Paratore A., Seneca R.P., Trask A. (1994). Decreasing the cost of trauma care: a system of secondary inhospital triage. Ann Emerg Med.

[41] Broadbent M., Moxham L., Dwyer T. (2007). The development and use of mental health triage scales in Australia. Int J Ment Health Nurs.

[42] Downey L.V.A., Zun L.S., Burke T. (2014). Comparison of emergency nurses association emergency severity triage and australian emergency mental health triage systems for the evaluation of psychiatric patients. J Ambul Care Manag.

