# Supplementary material for: Association Between Triage Score and Resource Use for Children Presenting to the Emergency Department with Behavioral Health Conditions
Source: J Am Coll Emerg Physicians Open. 2026 Sep 1;7(5):100490. doi: 10.1016/j.acepjo.2026.100490 (PMC13564129; doi:10.1016/j.acepjo.2026.100490)
Supplement: Supplementary file 1 [file mmc1.docx]

Table S1. Behavioral Health Reason for Visit Codes

| **Symptom** | **Reason for Visit**  **Codes** |
| --- | --- |
| Mood | 1100.0  1105.0  1110.0  1115.0  1120.0  1130.5 |
| Behavioral | 1125.0  1130.0  1130.1  1130.2  1130.3 |
| Substance Use | 1140.0  1145.0  1150.0  2320.0  2321.0  5910.0 |
| Psychosis | 1155.0  2305.0 |
| Suicidal Behavior | 5818.0  5820.0  5820.1 |
| Other | 1160.0  1165.0  2310.0  2315.0  2330.0  3130.0 |

Table S2. Definitions of Emergency Department Resources

| **Resources in Emergency**  **Severity Index Handbook** | **Corresponding Resource Code in National Hospital**  **Ambulatory Medical Care Survey** |
| --- | --- |
| Laboratory Test (1 point) | 1 if any of the following tests were obtained: [ABG],[BAC], [BMP], [BNP], [BUNCREAT], [CARDENZ],  [CBC], [CMP], [BLOODCX], [TRTCX], [URINECX], [WOUNDCX], [DDIMER], [ELECTROL], [GLUCOSE], [LACTATE], [LFT], [PTTINR], [OTHERBLD], [HIVTEST], [TOXSCREEN] |
| Electrocardiogram (1 point) | 1 if [EKG] |
| Radiograph (1 point | 1 if [X-RAY] |
| Advanced Imaging (1 point) | 1 if any of the following types of images were ordered: [CATSCAN], [CTCONRAST], [CTAP], [CTCHEST],  [CTHEAD]. [CTOTHER], [CTUNK], [MRI], [MRICONTRAST], [ULTRSND] |
| Intravenous Fluids (1 point) | 1 if [IVFLUIDS] |
| Nebulized Therapy (1 point) | 1 if [NEBUTHER] |
| Specialty Consultation (1 point) | 1 if [MHPROV] or [CONSULT] |
| Simple Procedure (1 point) | 1 if any of the following procedures were performed: [BLADCATH], [INCDRAIN], [LUMBAR], [SKINADH],  [SUTURE], [OTHPROC] |
| Complex Procedure (2 points) | 2 points if [GPMED#=1 or 2 = Ketamine] |

**Figure S1. Inclusion and Exclusion Population of Cohort**
